# Selections and Abstracts

**Published:** 1905-10

**Authors:** 


### SELECTIONS AND ABSTRACTS.

---

### THE TREATMENT OF DYSPEPSIA.

The *Clinical Journal* of May 10, 1905, contains the report of a lecture by LEONARD WILLIAMS in which he describes his methods. He says let us take a case of sthenic dyspepsia and see how it should be treated. We will assume the patient to be a man of middle age, who has at one time been fond of athletics, but who has been obliged by business exigencies to give them up, who is capable, hard-working, and energetic. He complains of epigastric discomfort after food, flatulent eructations, and mental irritability. The symptoms are not pronounced until some time has elapsed after a meal; indeed, he not infrequently associates them with the period before a meal, and may attribute them to hunger, a theory which obtains support from the fact that he is always better immediately after he has fully satisfied his rather vigorous appetite. He dines at 7:30 P. M., and is very often awakened between four and five in the morning with heartburn, pyrosis, sneezing, hiccoughing, asthmatic attacks, or other troubles, which however, rapidly subside as soon as he is able to "disperse the wind" of which his stomach appears to be full. In the day-time he is liable to suffer so much from palpitation that he feels sure there must be something wrong with his heart.

Now the first thing to be done with such a man is to clear his prima viae. Give him a dose of calomel (remembering that those of dark complexion bear this drug better than those who are fair); order him a Turkish bath, an electric light bath, or an ordinary hot bath, and induce him, if possible, to take some daily exercise in the open air, or at least at the open window. Warn him against wearing wool or flannel next his skin, and enjoin upon him the necessity for adequate mastication of his food. These and other warnings suggested by the special circumstances of the case must be emphasized; but the great, the paramount, the urgent need in such a patiet is for an antacid, to be taken either as soon as his symptoms commence, or if possible immediately before their onset.

The antacid which is most popular is the bicarbonate of sodium, but this salt is an antacid pure and simple, and is possessed of no sedative properties. It also has the disadvantage, especially where flatulence is troublesome, of increasing the amount of gas in the stomach. What is required is an antacid agent which is free from this objection, which at the same time is possessed of sedative properties. Such an agent is bismuth. There have been a great many differences of opinion regarding the merits of this drug, even so great an authority as Sir William Roberts going so far as to deny that it is an antacid at all. Sir Lauder Brunton, Dr. Burney Yeo, and other authorities, however, appear to esteem it very highly, and this view is supported by most of those who have made a systemic trial of its action. The disappointments attending its use in suitable cases have been almost certainly due to its employment in insufficient quantities. The ordinary B. P. doses are utterly useless; the minimum which the author employsis, of the subnitrate, 25 grains, and of the liquor bismuth ammoninio citratis, 2 drachms.

It is these two preparations which the author has learnt to appreciate very highly. The subnitrate may be given either in cachet form or suspended in a mixture. When prescribing it as a cachet he generally combines it with that excellent sedative oxalate of cerium, whose B. P. dose of 2 grains is also ridiculously inadequate; thus:

R. Bismuth, subnit., grs. xxv;
Cerii oxalat., grs. x.
M. Sig: Ter die post cib.

If, as is not infrequently the case, the patient has a gouty tendency, it is well to add five grains or a little more of pulvis guaiaci to each cachet, but in the author's experience the "little more" is very apt to produce griping, or purging, or both. Another drug which might be added to such a cachet is bicarbonate of sodium. It increases the alkalinity, but it increases also the bulk of the cachet and the quantity of gas in the stomach.

Although the subnitrate is frequently prescribed in a mixture (20 grains of the salt to 20 grains of pulvis tragacanth, compositus) it is not wise to do so. The carbonate acts nearly as well, and does not tend to decompose as the subnitrate does. On no account should the subnitrate be placed in a mixture with bicarbonate of sodium. The decomposition of the former leads to CO being evolved from the latter, and explosions are apt to occur.

If it is desired to give bismuth in a fluid form, the liquid bismuthi ammon. citratis should be used. It is the author's habit to combine it (as in the cachet) with a sedative—*i. e.*, hydrocyanic acid—thus:

R. Liq. bismuth, ammon. cit., f℥ij;
Syr. pruni. virg., f℥ij;
Aquae, q. s. ad f℥i;

This makes an agreeable and palatable mixture, but if with a view of correcting any gouty tendency say ℥ss tr. guaiaci ammon. is added it must be remembered to suspend the latter in 40 grains of mucilage of acacia, and even then the mixture will be deprived of its elegance. There is no objection to adding bicarbonate of sodium to this combination, but there is really no necessity to do so, for it is already sufficiently alkaline.

Now, whichever form is decided upon, the cachet or the mixture, it must be remembered that the proper time for its administration is some time after food. The length of time which should be allowed to elapse between the meal and the taking of the remedy depends of course, upon the size of the meal. A full meal will take five hours to digest and will use up a great deal of HCL. A light meal, especially if it be poor in proteids, will use up very little acid—that is why sthenic dyspepsia is so much more common after light meals—and the surplus will want neutralizing relatively soon. It will want neutralizing sooner after breakfast than after luncheon, and sooner after tea than either. After a full dinner the symptoms frequently do not show themselves until about 4 or 5 A. M., and may then, in addition to pyrosis and heartburn, take the far more obscure forms of hiccoughing, sneezing, asthmatic and even anginal attacks. The tendency of any symptoms, however little connected with the stomach they may at first sight appear, to recur regularly at 4 or 5 A. M. should give rise to a suspicion that dyspepsia is at the root of the mischief. It is probable that much of the success which has attended the practice of giving alkalies before meals has been due to the fact that the period immediately preceding one meal is the period which witnesses the close of the digestion of the last—the period, that is, in which there is surplus acid waiting to be neutralized. However that may be, there can be do doubt that the administration of alkalies, and especially of bismuth, at a suitable interval after food offers a means of relief in sthenic cases which is practically unfailing, and the writer would even go as far as to say that if relief is not obtained by such means, then the case is certainly not a dyspepsia of the class under consideration.

---

### ON THE IMPOSSIBILITY OF DIFFERENTIATING SO-CALLED "PARATYPHOID" FROM TYPHOID FEVER EXCEPT BY A BACTERIOLOGICAL EXAMINATION OF THE BLOOD. *

BY WARREN COLEMAN, M.D., NEW YORK.

If cases of infection by intermediate members of the typhoid-colon group of bacilli, when pursuing a typhoidal course, can not be distinguished from typhoid fever without the aid of laboratory methods, the introduction of the term "paratyphoid fever" is clearly unjustifiable. I shall endeavor to prove that so-called paratyphoid and typhoid fever are identical clinically, and that they can be differentiated only by the recovery of the bacillus concerned from the blood.

Schottmüller introduced the term "paratyphoid fever" in 1901. In 1902, in a paper entitled "Types of Infection Produced in Man by Intermediate Members of the Typhoid-Colon Group of Bacilli," † I took the ground "that paratyphoid infections do not constitute a clinical entity; that there is as great diversity among the different types of typhoid fever, as between typhoid fever and paratyphoid infections; that the idea of the specificity of typhoid fever should be abandoned, and that the scope of its etiology should

* Read at a meeting of Section on Medicine, New York Academy of Medicine, April 18, 1905.

† Am. Med., 1902, Sept. and Oct.

be broadened to include *Bacillus alkalingenes fœcalis*, *Bacillus typhosus*, *Bacillus paratyphosus* and certain members of the paracolon group." At the time this paper was written only about fifty cases of paratyphoid infection had been published. As the number of cases increased and the subject has become better known, I have not only seen no reason to modify the above view, but have become more thoroughly convinced of its correctness.

A brief review of the bacilli constituting the typhoid-colon group will enable me to explain my conception of the subject more clearly. I shall consider only the bacilli pathogenic for man. Petruschky's *Bacillus alkaligenes fœcalis,* which lies just without the group on the typhoid side, must be included, because it has produced typhoidal symptoms. The other members of the group are *Bacillus typhosus,* the paratyphoid bacilli which are not identical either in biological or serum reactions, the paracolon bacilli, which likewise differ among themselves, *Bacillus enteritidis,* consisting of different strains, *Bacillus ptsitacosis* and *Bacillus coli communis.*

All of these bacilli have produced diseases characterized by typhoidal symptoms, with the possible exception of *Bacillus coli communis.* I grant at once that infections by these different organisms are distinct diseases *from the bacteriologist's standpoint.* But bacteriological are not necessarily clinical entities. Pneumonia and infective endocarditis are examples. Further, if clinical distinctions are to be made on the basis of biological differences in organisms, we must be consistent, and give a name to each subvariety of infection. It needs but a moment's reflection to see where this policy would lead us in the typhoid-colon infections.

The pathological anatomy of the cases of infection by the intermediates which have come to autopsy has differed from that of typhoid fever. Peyer's patches have escaped, though four of six cases showed intestinal ulcers. What the ultimate significance of these facts will be, I think no one can tell. But since the number of cases studied is so small, it will clearly be wiser not to draw conclusions from them.

*The Clinical Diagnosis of Typhoid Fever and Paratyphoid Infections: Twenty-one-Day Cases.*—A positive clinical diagnosis of even a typical case of typhoid fever is beset with difficulty and is generally made after the fact. No case is typical, until the disease has finished its course. More than once has general miliary tuberculosis been mistaken for typhoid fever. (I do not, of course, refer to the probable diagnosis of the usual case. We are dealing with *exact*, not probable, diagnosis.)

Some of the reported cases of paratyphoid infection have been described as typical attacks of typhoid fever. Some have been mild, some severe. Among them they have presented all the symptoms and many of the complications of typhoid fever. There has been no symptom of typhoid fever constantly absent. There has been no symptom constantly present that we may not get in typhoid fever. All that the most ardent advocates of the use of the term "paratyphoid fever" claim for it as a distinct disease is, that the cases are milder, that they are of shorter duration (I shall consider the cases of less than twenty-one days' duration later), that the mortality is lower, and that some complications, *e. g.*, myositis and purulent arthritis, are more frequent. But it must be borne in mind that these conclusions have been drawn from less than two hundred cases, and that the majority of the cases reported as infections by an intermediate have not been subjected to the only final diagnostic criterion—the recovery of the bacillus from the blood. But even admitting that all the cases reported as such were infections by intermediates, I claim that one would find as many statistical discrepancies among an equal number of cases of typhoid fever taken at random.

My personal experience is limited to one case. But that case passed for a typical, mild case of typhoid fever, until Buxton found that the bacillus we had isolated from the blood gave the cultural reactions of paratyphoid bacilli.

*Short-Duration Cases.*—If the *final* diagnosis of a typical case of typhoid fever is difficult, what shall be said of the diagnosis of the irregular cases? Witness the former elaborate discussions on "simple continued fever." I have no hesitation in saying that I believe the majority of these cases to have been caused by some member of the typhoid-colon group of bacilli. The time has even come when we must take into consideration the possibility of the occur rence of primary systemic infection by the colon bacillus itself. The literature upon the subject is slowly accumulating. Buxton and I have had one case which did not give the typhoid serum-reaction, and from the patient's blood we isolated *Bacillus coli communis* in pure culture.

The argument that the cases of so-called "paratyphoid fever" have been of shorter duration than typhoid will not serve to distinguish them clinically. During the past summer I have had ample proof of this at Bellevue Hospital. I do not recall having seen in any one season so many cases of short-duration, and otherwise irregular, typhoid. The diagnosis in some of them was placed beyond dispute by the recovery of typical typhoid bacilli from their blood. One lasted ten days, another fourteen, and their symptoms corresponded perfectly with those of some of the reported cases of "paratyphoid fever." These cases show, then, that short duration is not distinctive of infections by the intermediates.

*Serum Reaction.*—By way of differentation, certain authors have attributed much diagnostic significance to absence of the serum reaction against *Bacillus typhosus.* Even positive diagnoses of paratyphoid infections have been made upon such negative evidence. But a negative serum reaction in a case of suspected infection by some member of the typhoid-colon group means one thing only—that the agglutinins have not developed, or at least not in sufficient quantity to give a reaction. Variability in the time of appearance of the reaction is well known. I have seen it delayed to the seventy-first day of an irregular case.

Even a positive reaction with a paratyphoid bacillus at higher dilution than with *Bacillus typhosus* does not prove that this bacillus is the cause of the infection. Grünbaum and Jolly ‡ have recently (January) published a paper on the inconclusive nature of the results of the agglutination reaction in cases of typhoid fever. They tested the reactions of the blood of positive typhoid cases (proven by bacteriological examination of the blood) against paratyphoid bacilli and obtained agglutination in 70 per cent. of the cases examined. In some cases the paratyphoid bacilli were agglutinated

‡ Münch. med. Woch., 1905, Jan. 17.

at higher dilution than typhoid bacilli. In 55 per cent. of the cases a colon bacilli reacted with the sera, *Bacillus enteritidis* (Gärtner) in 22 per cent.

This only confirms what Durham demonstrated some years ago that the reactions of the typhoid-colon group are special, but not specific. The various members of the group give a group reaction, that is, any immune serum may react with several or more members of the group at low dilutions (frequently at 1 : 10 to 1 : 40, more rare at 1:1000).

It is, therefore, necessary to exercise caution in drawing conclusions even from positive reactions. If a serum reacts at 1 : 1000 with a paratyphoid bacillus and at 1 : 100 or less with *Bacillus typhosus,* the case is probably, but not certainly, one of paratyphoid infection. However, a group reaction at 1 : 20 is sufficient for practical purposes. Prophylaxis and treatment are the same in any typhoidal case of infection by any member of the group. And it can not be too strongly urged that the diagnosis of paratyphoid infections concerns the bacteriologist more than the practicing physician.

A successful bacteriological examination of the blood, with proper identification of the bacillus recovered, becomes then the only final criterion of diagnosis in the differentiation of infections by various members of the typhoid-colon group of bacilli. (This statement, of course, does not refer to cases of infection by Gärtner's *Bacillus enteritidis,* except when they take the typhoid form.)

In further substantiation of my position I would call attention to the fact that the term "paratyphoid fever" has been discredited in Germany, Belgium and Russia.

*Conclusions.*—(1) That paratyphoid infections can not be distinguished from typhoid fever except by the recovery from the blood of the bacillus concerned and its proper identification. (2) That the present state of our knowledge makes it advisable to consider typhoid fever clinically as a disease which may be caused by several members of the typhoid-colon group of bacilli. (3) That the term "paratyphoid fever" is not only unnecessary, but misleading.

For the complete bibliography of paratyphoid infections, see Vallet, Bull. med., Par. 1905, XIX, 33. Jürgens, Zeitschr. f. klin. Med., Vol. XXV, Nos. 1 and 2.

### THE DIAGNOSIS AND TREATMENT OF FRACTURE OF THE CARPAL SCAPHOID AND DISLOCATION OF THE SEMILUNAR BONE, WITH REPORT OF THIRTY CASES.

Cadman and Chase in *Annals of Surgery,* March and June, 1905, urge greater care in the diagnosis of injuries of the wrist, and in closing their extensive paper they summarize the following points:

1. Sprains of the wrist which do not promptly recover are in many cases fractures or dislocations of the carpal bones.

2. The large majority of such injuries are either simple fractures of the scaphoid or anterior dislocations of the semilunar bone.

3. These two injuries are frequently combined, and in such cases the proximal fragment of the scaphoid is usually dislocated forward with the semilunar.

4. Simple fracture of the scaphoid gives a definite picture and may be recognized, even without the X-ray, by association of the following symptoms, viz.: (a) The history of a fall on the hand. (b) Localized swelling in the radial half of the wrist-joint. (c) Acute tenderness in the anatomical snuff-box when the hand is adducted. (d) Limitations of extension by muscular spasm the overcoming of which by force causes unbearable pain.

5. A broken scaphoid has little power of repair and appears capable of but little callous formation.

6. Fractures of the scaphoid which remain untreated or are treated by massage and active and passive motion generally, if not always, remain ununited, and the original symptoms persist for years with only slightly abated intensity.

7. Cases of fracture of the scaphoid may unite, if the motion of the wrist is prevented during the first four weeks after the injury, but if by this time no union has occurred future union is unlikely.

8. Excision of the proximal half of a fractured scaphoid gives a somewhat better result than conservative treatment.

9. A posterior incision to the outer side of the tendons of the extensor communis digitorum gives an easy and safe access to the proximal half of the scaphoid.

10. Passive motion of the wrist-joint and active motion of the fingers should be begun within a week after this operation.

11. The possibility of a bipartate scaphoid should be considered in interpreting X-rays of simple fractures of the scaphoid, but its occurrence must be very rare in comparison with fracture.

12. Anterior dislocation of the semilunar bone should be recognized clinically, even if without X-rays, by the association of the following symptoms, viz.: (a) The history of an injury of considerable violence to the extended or twisted wrist. (b) A silver fork deformity, the posterior prominence of which corresponds with the head of the os magnum, and between which and the lower end of the radius is found a groove representing the position formerly occupied by the now anteriorly dislocated semilunar. (c) A tumor under the flexor tendons of the wrist just anterior to the lower end of the radius. (d) A shortened appearance of the palm as compared with the other hand. (e) Stiffness of the partially flexed fingers, motion of which either passive or active is painful. (f) The persistence of the normal relation of the styloid processes of the radius and ulna and the existence of shortening from the radial styloid to the base of the first metacarpal.

13. Recent dislocations of the semilunar may be reduced with good results, even after the fifth week, by hyperextension followed by hyperflexion over the thumbs of an assistant held firmly in the flexure of the wrist on the semilunar.

14. Irreducible dislocations demand excision of the semilunar and the whole or a portion of the scaphoid, if there is coincident fracture of the latter.

---

### ELASTIC CAPSULE MEDICATION.

Eli Lilly & Company have just issued a handsome illustrated pamphlet on Elastic Capsule Medication, giving in natural colors the important preparations of this class. The work is replete with concise therapeutic notes on the various oils and oleoresins used in modern medical practice and the forms in which they may be administered best. The book is addressed to the practitioner and will be found full of useful information on this important and increasing line of recent pharmaceuticals; a copy will be sent to any practitioner on application to the publishers—Eli Lilly & Company, Pharmaceutical Chemists, Indianapolis.

### THE MEANING OF SUBSTITUTION TO THE PHYSICIAN.

The substitutor prescribes for your patient without regard to your reputation or the welfare of your patient, assuming that you do not know your business.

Why does he do it? For illegitimate profit.

What are you going to do about it?

The substitutor.—You lose your patient but don't know why.

The substitutor.—The man who sells your patient a gold brick. Your patient believes you did it.

The name of the physician who permits substitution on his prescription—E. Z. Mark, M.D.

The substitutor.—The man without originality or initiative. He wants to degrade you. Will you permit it?

The substitutor.—The man who sacrifices you and your patient to satisfy his avarice.

What are you going to do about it?

The substitutor.—Ananias was an angel compared to him. The first stole money and then lied about it. Penalty: Death. The substitutor steals your patient's money, his chance for life and your reputation as well. Penalty: Increased bank account.

---

### HOW TO GIVE LECITHIN.

When organic phosphorus is indicated in general debility and malnutrition, lecithin derived from the yolks of eggs presents it in a highly available form. Many of the preparations of lecithin are nauseating and repulsive, especially to sensitive stomachs. In giving lecithin it is entirely unnecessary to offend the palate and disgust the patient with a nauseous dose. The best way for its exhibition is in the form of Lilly's granular lecithin, in which the lecithin is associated with sugar and chocolate. Sixty grains of the granular lecithin contain one grain of the chemically pure substance. Its pleasant flavor renders granular lecithin particularly acceptable to small children. In cases where sugar is contra-indicated, lecithin may be given associated with pure egg oil in the form of Lilly's gelatin globules of lecithin; the egg oil, like the lecithin, being derived from the yolks of fresh eggs. Samples of these fine preparations may be had by addressing Eli Lilly & Company, Indianapolis.

### THE ELIMINATION OF THE MOSQUITO.*

ALVAH H. DOTY, M.D., NEW YORK CITY.

I am quite sure that the members of the American Medical Association are familiar with the general literature regarding mosquitoes, and that a detailed account of the different varieties of these insects, their anatomy, method of development and the physiologic reason why they transmit disease is unnecessary. I have chosen, therefore, to present some practical facts which have a direct bearing on the work of exterminating the mosquito.

A decided and very satisfactory change has taken place in the attitude of the public toward this subject during the past three or four years. At first, efforts to exterminate the mosquito in this country were looked on mainly from a humorous point of view, and but little co-operation was extended by the laity. The public, however, has learned that the object of this work is not only to prevent the annoyance which the bite of this insect inflicts, but also to prevent the transmission of the disease, inasmuch as it has been proved that yellow fever is transmitted by the variety of mosquito known as the stegomyia, and malarial fever by the anopheles, and that so far as we know at the present time this is the only means by which these diseases are propagated.

In the extermination of the mosquito, it is imperative that we should be familiar with certain habits or conditions of this insect, otherwise our efforts are not apt to be attended with success. We must know, first of all, that mosquitoes propagate only in water. The importance of this fact is that it gives definite information regarding the situation of breeding places. We know that the eggs deposited on the surface of the water develop into the larvæ, commonly known as the "wigglers," then into the very transient stage of the pupæ, which in appearance may be regarded as a change in the shape of the larvæ, and that these are very soon transformed into the winged insects, and that from the deposit of the eggs to the development of the winged insect only from twelve to twenty-five days elapse. The latter period, as a rule, is required for the

*This paper was prepared for the Section on Hygiene and Sanitary Science, but was not read, owing to the absence of the author.

development of the anopheles or malarial mosquito. So far as my own investigations show, the propagation of the mosquito is, as a rule, most active in stagnant or offensive waters, or in that which contains considerable organic matter. Two instances have recently come under my observation where broken sewer pipes discharged into ground depressions, causing collections of offensive water. In the immediate vicinity of these were pools of water which were not contaminated; i. e., collections of rain water and not of offensive fluid. In both instances it was found that the contaminated water soon became exceedingly rich in mosquito larvæ, whereas in the other pools, within two or three hundred feet distant, which contained comparatively clear water, there were very few, if any, larvæ found.

Experiments made at the New York Quarantine Station have practically corroborated this. In these experiments large superficial wooden tanks were employed, in which different kinds of water were placed; i. e., rain water, hydrant water, water in which decomposed meat and fish had been placed, and water containing other offensive material. These tanks were exposed, side by side, in a place infested with mosquitoes. In all the tests, it was shown that the deposit of mosquito eggs was always greater in the tanks containing the offensive water. Experiments on a smaller scale were also made. Exposed pails and glasses containing the different kinds of water already referred to were employed and the same results were noticed, viz., the deposit of eggs was much greater in offensive water. These experiments were repeated many times with the same result, and fully confirmed the conditions which I have found in mosquito-breeding districts. The experiments to which I have just referred were made with the culex, which is the commonest type of mosquito. There is, however, a variety of the culex known as the *Culex solicitans*, the striped-legged, Atlantic coast or salt marsh mosquito, which may be excepted from the above, inasmuch as it breeds in salt water or in salt or brackish marshes. Furthermore, the malarial mosquito, known as the anopheles, while it may be found breeding in offensive water, prefers small pools of water which are not so contaminated and which may be found in excavations containing surface water frequently having a green scum on the surface. I have found the anopheles larvæ in water contained in small ground depressions along the edge of swamps, also in unused receptacles which are found about houses. Occasionally the anopheles larvæ may be found in the same breeding pool with the culex; this, however, is not common. In a general way, it may be said that mosquitoes will breed in almost any receptacle and in the most unusual and unsuspected places, commonly in rain-water barrels, cisterns, cesspools, tin cans and other metal, glass and wooden receptacles, roof leaders, crotches of trees, etc. It is very necessary that physicians should be familiar with this fact.

My experiments show that in captivity the *Culex solicitans* will sometimes deposit their eggs on fresh water contained in glass jars; whether or not tney will do this if they are free has not yet been fully decided. It is probable that along the Atlantic coast the salt marshes in many places furnish fully eighty per cent. of the mosquitoes in these sections. Dr. John B. Smith, State Entomologist of New Jersey, who has carefully investigated this variety of mosquito, states that it will deposit its eggs on the soft earth, in salt marshes along the sea coast, and that these eggs will remain intact for months until the swamps overflow and there is sufficient water to develop them into larvæ and subsequently into the winged insects. My experiments have partially confirmed this. I have confined the Atlantic coast or striped-legged mosquitoes in glass jars, in which was placed soft earth taken from salt marshes along the coast, and it was soon found that the eggs were deposited on this material. When salt water was added to this, after the eggs had been laid a number of days, the larvæ very promptly appeared. No proof, however, has ever been produced to show that the larvæ can be developed from these eggs without the presence of water. If the *Culex solicitans* be confined in water to which various amounts of salt have been added, or in jars containing sea water, a deposit of eggs on the surface will soon be noticed; however, I have been unable to secure this result with any but the Atlantic coast mosquito.

A general impression exists that the life of the mosquito is very short, probably a few days only. While it is true that the mosquito is not a resistant insect and easily succumbs to all sorts of influences, it is a fact that it lives indefinitely. In the hibernating stage it is known that it may live from the fall to the following spring, and during the warm weather, or in its active stage, it may live a number of weeks. It is practically impossible to arrive at any definite conclusion regarding the life cycle of the mosquito, inasmuch as experiments do not furnish satisfactory evidence, for the reason that they can not be carried out under the same conditions that naturally exist. In other words, mosquitoes may die from captivity alone, or when they are unable to select the character of food which they require. It is safe to assume, however, that if they will live a certain period in captivity, they will live equally long when they are free to go and come as they please. There have been many investigations made and published on this point, and experiments have shown that in captivity mosquitoes will live for a number of weeks. I have kept them in wire cages containing nothing but sugar water for almost two months; furthermore, mosquitoes may be kept for a long period in captivity without food or water, provided sufficient air be present. Aside from the interest in securing definite knowledge in this direction, the length of time which mosquitoes may live in captivity has an important practical bearing, as it has been claimed that yellow fever may be transmitted from one place to another through the agency of the stegomyia, or yellow fever mosquito, confined in baggage, etc. In order to secure so far as possible definite information regarding this part of the subject, I carried out the following experiments: A number of boxes which could be compared to trunks, also canvas bags, such as sailors use to contain their clothing, and other receptacles were filled with clothing, blankets, bedding, etc. Mosquitoes were collected from a house stationed in a thickly-infested mosquito district. This was done by placing glass test tubes over the mosquitoes. When a sufficient number were secured the openings of the tubes were filled with light cotton plugs. The tubes were then introduced into the receptacles already described, the cotton plugs removed and the tubes gradually withdrawn and shaken sufficiently to drive out the mosquito. This method was employed in order to prevent any possible injury to the insect. In each experiment a record was kept of the number of mosquitoes in the different receptacles, and, although many experiments were made, it was found that in no instance did the mosquitoes in any receptacle live longer than thirty hours. I believe, therefore, that the treatment of baggage coming from yellow fever ports is unnecessary, provided the period of transit exceeds two days.

Considerable discussion has taken place among those who have investigated the mosquito regarding the origin of the first crop of these insects, which appear in the spring or summer of each year. As I have already stated, Dr. John B. Smith claims that the egg of the *Culex solicitans*, or striped-legged or Atlantic coast mosquito, may be deposited on the moist earth, in brackish or salt-water swamps, and that in this situation, he believes, they will remain dormant or hibernate until the following spring, when an overflow of water takes place and the eggs become active and are developed into lavæ and subsequently into the winged insects. It has also been shown that larvæ will hibernate and that during the winter they may be found in ice and after being thawed out become active. The latter phenomena has come under my notice, but I have seen no instance in which larvæ after being frozen and thawed out have developed into winged insects. This may occur, but I am sure that it is uncommon. Allthough the eggs of the *Culex solicitans*, Atlantic coast or striped-legged mosquitoes, may hibernate during the winter, and be the origin of the first crop of this variety of the mosquito in the spring or summer, I am satisfied from my own experiments that the first crop of mosquitoes which come to us each year, particularly in inland towns, or about fresh water supplies, is due to the hibernation of the winged insect. It is common experience with all who live in mosquito-infested districts frequently to find these insects in midwinter, particularly in overheated rooms, etc. It is evident that when cold weather approaches the mosquito instinctively seeks some protected place, such as crevices in the interior of tree stumps, cellars, barns, interior of houses, etc., and, while there are probably but comparatively few which do not succumb to the cold weather, sufficient survive to perpetuate the species during the following year.

Another very much disputed point regarding the mosquito is whether or not they go far from their breeding-places. While there is indisputable evidence that mosquitoes at times are found far from home, there is also the best evidence that they do not go willingly. One of the most satisfactory instances that I know of, which proves that they are found some distance from their breeding-place is that furnished by the New York harbor pilots, whose boats are frequently anchored along the Jersey coast, three or four miles from land—occasionally they become suddenly infested with mosquitoes. The pilots are unanimous in their statement that when this occurs there is always a breeze from the shore. While indisputable evidence has been presented to show that mosquitoes go far from home on land, as well as at sea, there is good reason to believe that this does not commonly occur. It is the opinion of all who have carefully studied this insect that they do not willingly go far away, and I believe that we are justified in assuming that if a section of country be infested with mosquitoes a breeding-place exists in the immediate vicinity. I speak emphatically in this matter, as many times after it has been positively stated that mosquitoes must have come a long distance, it has been found that breeding-places existed in the neighborhood, and there is no doubt that the difference of opinion which existed on this subject is largely due to the fact that local breeding-places are frequently overlooked. When it is remembered that the female mosquito will lay three or four hundred eggs at one time, and that it only requires from twelve to twenty-five days for those eggs to develop into the full-grown winged insect, it will be better understood how a small receptacle will breed a large number of mosquitoes. Therefore, before hunting for the breeding-places of mosquitoes at some distance from home, we should first examine every detail of our own premises to ascertain if receptacles for the propagation of this insect can not be found.

These breeding places are often discovered in the most unsuspected locations. In connection with this and as an item of interest, I will refer to an investigation made two or three years ago. My attention was called to a number of cases of malarial fever occurring in two or three houses adjoining each other. Anopheles were found in the bedrooms of these patients during the evening.

In close proximity to these houses were found numerous pools of water, and, although each one was examined with great care, no anopheles larvæ were found. The most careful inspection was made of every cistern, rain-water barrel, etc., in the neighborhood with the same result. This lent some color to the belief that the mosquito in this instance might have come from a distance. Further investigation was made, however, and in the garden of a house in the rear of those containing the cases of malarial fever, and which had been vacated for some time, was found an old cake pan almost entirely covered by long grass, and in this was found a large number of anopheles larvæ. An ordinary inspection would not have discovered this receptacle.

As a resume of what we know at the present time, it may be said:

First, mosquitoes do not propagate without water, and, as a rule, so far as the culex is concerned, the more offensive the water, the greater the propagation. Although the anopheles or malarial mosquito will propagate in offensive water, sewerage, etc., they are more apt to be found in small collections of water, about fresh water swamps or in small receptacles about the house. In large ponds or pools of fresh water, particularly those which contain small fish, which prey on the larvæ, mosquitoes of any variety are not usually propagated, but the little pockets of water along the edges of these ponds or in other places frequently act as breeding places. We also know that a variety of mosquitoes known as the *Culex solicitans*, or the striped legged, Atlantic coast or salt marsh mosquitoes, breeds in salt water or in salt marshes. Mosquitoes breed in cisterns, rain-water barrels and cesspools about the premises, no matter how small or where they are situated, may also act as breeding places. It is about our own homes, therefore, that the examination should first be made to ascertain where the propagation of this insect is carried on.

Second, the life of the mosquito is not confined to a few days, but under various circumstances may extend over a period of weeks or months.

Third, the first crop of mosquitoes which appears in the early summer of each year, particularly in inland towns, is principally due to the deposit of eggs which have hibernated during the winter months. These, of course, are comparatively few in number, but are sufficient for the purpose above referred to. The variety of the mosquito known as the *Culex solicitans* is probably largely perpetuated by the hibernation of eggs, which are deposited in the soft earth, in salt marshes along the sea coast, and remain dormant during the winter; when the warm weather returns and the marshes are overflowed with water, these eggs become active and develop into larvæ; subsequently into winged insects.

Fourth, although there is conclusive evidence that mosquitoes are sometimes carried long distances from home, they do not willingly go far from their breeding places, and it may be assumed that if a section is infected with mosquitoes breeding places exist in the immediate vicinity. It is probable that an exception may be made in the case of the *Culex solicitans*, or salt-water mosquito, which appear to go further from home than other varieties of the culex or the anopheles. There is good reason to believe, however, that they do not breed away from the salt marshes or in collections of fresh water.

Efforts to prevent the propagation of the mosquito consist in abolishing or removing receptacles which contain water. This applies to both large and small ground depressions, swamps, etc., and to portable and stationary receptacles about buildings. The scientific, practical and radical method of removing water in ground depressions is by drainage or filling in, and the use of petroleum oil in these instances can not be regarded as a substitute for this purpose and should only be used as a temporary measure. In mosquito-infested districts our first action should be to remove, so far as possible, from dwelling-houses and other buildings, all sorts of metal, glass and wooden receptacles for water. Cisterns and rain-water barrels should be supplied with tight-fitting covers; by having the center of these covers constructed of wire netting sufficient air is admitted. Roof leaders should be kept properly graded; otherwise parts of them may act as breeding places for the mosquito. If ground depressions either about the premises or in the neighborhood can not be drained or filled in, petroleum oil may be used as a temporary measure.

The crude petroleum is probably superior to the refined oil and should be used in the proportion of one pint of oil to a water surface of about twenty feet in diameter—even a less amount of oil may be effective. This procedure should be repeated every two weeks. The method by which the oil destroys the larvæ or wigglers is probably not by a toxic effect, but by a mechanical one. The larvæ must come to the surface of the water for air at least every two minutes. If watched carefully it will be seen that the culex, or common variety, approach the surface of the water at right angles, with the tail uppermost, the air being taken in at this end, which is extended sufficiently above the surface. The anopheles larvæ may be distinguished from the culex at this stage, inasmuch as they do not lie at right angles to the surface of the water, but parallel to it, and receive the air by projecting the head above the water instead of the tail. It is probable that the oil thrown over the surface plugs up the delicate respiratory apparatus of the larvæ and practically suffocates them. This is largely corroborated by experiments. If a small amount of petroleum oil is thrown over the surface of the water in a glass jar containing larvæ, the latter will succumb in from ten to twenty minutes; toxic agents do not kill them with such rapidity, as careful investigation has proved. The odor of petroleum oil is either very unpleasant or injurious to the winged insect. It has frequently been noticed when petroleum has been used in swamps in large quantities that the number of mosquitoes abruptly diminishes.

In treating large bodies of water with petroleum, the ordinary garden sprinkling-pot is a good and practical method of distributing it. Experiments made with permanganate of potassium, bichlorid of mercury, sulphate of copper, carbolic acid, etc., have shown that these agents are greatly inferior to petroleum for this purpose. Their action is slow, and the mosquito larvæ live in comparatively strong solutions. For instance, larvæ have remained active from one to three days in a 1/1500 solution of bichloride of mercury. Even comparatively strong solutions of carbolic acid or permanganate of potassium do not destroy them for some time. In some very exhaustive experiments made with sulphate of copper and lime for the destruction of mosquito larvæ,† I found that these agents did not destroy the mosquito by a toxic effect, but slowly by clarifying the water and precipitating the organic matter which it contained, thereby removing the nourishment from the larvæ. Furthermore, it must be remembered that pools of water throughout the country may be used for drinking purposes, particularly by animals, and that the use of such agents as bichlorid of mercury, carbolic acid, etc., are, therefore, unsafe. On the other hand, the petroleum oil is cheap, practically harmless and destroys the larvæ at once, and so far as we know at the present time is superior to anything else for this purpose, provided proper drainage or filling in can not be effected.

All varieties of mosquitoes are most active after sundown. This we know, not only from the annoyance which they cause us, but from the indisputable testimony presented by medical experiments with the malarial and yellow fever variety. During the day, when the mosquitoes are generally inactive, they select for their abiding place the high grass and underbrush. Therefore, this material should be removed if present about the premises in mosquito-infected districts.

The points to which I have referred in this paper constitute the bulk of what is at present really known regarding the habits of the mosquito and the means of exterminating them. Many investigations, however, are now in progress in this country, and there is good reason to believe that much valuable information will be added to our knowledge during the next year.

---

### INTESTINAL DISORDERS DUE TO LACK OF NORMAL INTESTINAL ALKALINES.

At this period of the year we are called upon to consider carefully the application of therapeutic measures to be adopted in the treatment of disease of the gastro-intestinal tract. Conditions met in these cases have a marked degree of similarity, due primarily to a faulty or altered secretion of intestinal juices, and secondarily through errors of diet, etc. The contents of the bowel are found

† Medical Record, Jan. 25, 1905.

to be made up of a fermenting mass of decomposed food, broken-down mucous membrane, together with fluids of intensely acid reaction loaded with pathogenic bacteria. The logical treatment indicated would call for a prompt removal of the source of infection and the restoration of normal secretion.

A prominent practitioner in the South, whose wide experience justifies authority, recently embodied in a paper the following statement: "In diseases of the intestinal tract in children or adults, whatever the diagnosis may be, we always trace the origin of the trouble to a *want of alkalines* to correct an excess of acidity during the digestive process." This is of interest to us, as it gives the key to the marked results following the administration of the alkaline antiseptic, Glyco-Thymoline, which not only corrects existing hyperacidity with its concomitant symptoms, but causes by its exosmotic property a rapid depletion of the engorged membrane and a stimulation of the glandular system to normality, whereby the proper amount of alkaline fluids will be secreted. Therefore, it is well to remember that Glyco-Thymoline not only corrects the effects of disease, but aims to reestablish those processes of digestion and assimilation which are wanting.

In severe cases of cholera infantum, dysentery, ileo-colitis, etc., the solution should be administered as a colon flush, using a ten per cent. solution of about 100°F. This treatment combined with 3i to 3ii doses per oram serves to rapidly eliminate all toxins, promote an aseptic condition of the bowel and to encourage what is most needed—the secretion of normal alkalines.

---

### A MISERABLE OUTRAGE.

The depths of venality to which the nostrum-makers descend in order to gather in a few more dollars are beyond the belief of those possessing normal human sympathy. One of the worst recent instances of this sort of inhumanity is now to be found in some newspapers of the Southern States. Inserted as news, not as an advertisement (*vide*, for example, the Shreveport, (La.) *Times* of August 16, 1905) appears what is called "an interview with Dr. Hartman concerning the yellow plague." Dr. Hartman needs no introduction, being widely known to physicians as that citizen o Columbus, Ohio, who has amassed a colossal fortune by selling to a gullible public as a " medicine, " under the name of peruna, low-grade whisky flavored with cheap bitters. Aided by two or three avaricious renegade physicians, he has enmeshed in his fraud thousands of clergymen, lawyers, politicians, etc., and, if what is reported is true, has undoubtedly created an appetite for intoxicants in unsuspecting men and women and in innocent children. In the present instance, the fraud on humanity is at least equal in perfidy to that of the traitor who sells out his country. A summary of this interview is being spread broadcast over a portion of the United States for the benefit of yellow-fever sufferers. "In all such cases it is advisable for the person to take peruna." " Begin taking peruna at once in teaspoonful doses so as to harden and heal the mucous membrane against the possible invasion of yellow fever poison."(!) " Peruna should be used during the whole course of the epidemic." "I feel sure that any person following this advice is in no danger of taking yellow fever." What could be worse? Shall such things be permitted to continue? Can nothing be done to protect a fear-stricken people from such damnable outrages on humanity? And what about newspapers that will join in a conspiracy of this kind to defraud their readers for a few paltry dollars?—*Editorial, Journal A. M. A.*, Sept. 2, 1905.

---

COLOBEPATOPEXY OR COLON SUBSTITUTION.

E. W. Andrews presents a new operation for adhesions after gall-stone operations and concludes as follows:

1. Gall-tract adhesions are inevitable after disease and operation.
2. They are beneficent, harmless and symptomless in all but a few cases.
3. These few represent malposition rather than trouble from adhesions *per se*.
4. The colon, gall bladder, duodenum and pylorus can adhere to each other without impairing their function. The other parts of the stomach cause trouble if involved.
5. Such adhesions will reform when separated, unless the colon be substituted for the stomach.
6. The causing of colon adhesion to the liver does not disturb its function perceptibly.
7. Probably certain vague gastric disturbances have been treated by gastroenterostomy when the patients would have had more benefit from this operation.

